# Cardiovascular MRI–based Biventricular Perfusion Assessment in Two Patients with Chronic Thromboembolic Pulmonary Hypertension Undergoing Pulmonary Thromboendarterectomy

**DOI:** 10.1148/ryct.250296

**Published:** 2025-11-20

**Authors:** Lexiaozi Fan, María Davó-Jiménez, Brandon C. Benefield, Nour Beydoun, David Dushfunian, Sebastian Cohn, Michael Cuttica, Ruben Mylvaganam, S. Chris Malaisrie, Stephen Chiu, Li-Yueh Hsu, Ryan Avery, Daniel Schimmel, Yasmin Raza, Jordyn Durkin, Donny Nieto, Michael Markl, Daniel C. Lee, Daniel Kim, Benjamin H. Freed

**Affiliations:** ^1^Department of Radiology, Northwestern University Feinberg School of Medicine, 737 N Michigan Ave, Ste 1600, Chicago, IL 60611; ^2^Division of Cardiology, Department of Internal Medicine, Northwestern University Feinberg School of Medicine, Chicago, Ill; ^3^Department of Biomedical Engineering, Northwestern University, Evanston, Ill; ^4^Division of Pulmonary and Critical Care Medicine, Northwestern University Feinberg School of Medicine, Chicago, Ill; ^5^Division of Cardiac Surgery, Northwestern University Feinberg School of Medicine, Chicago, Ill; ^6^Department of Radiology and Imaging Sciences, National Institutes of Health, Bethesda, Md

**Keywords:** Cardiac, Pulmonary Arteries, Chronic Thromboembolic Pulmonary Hypertension, Pulmonary Thromboendarterectomy, Quantitative Perfusion Cardiovascular MRI, Myocardial Blood Flow, Myocardial Perfusion Reserve

## Abstract

Chronic thromboembolic pulmonary hypertension (CTEPH) can lead to right ventricular (RV) ischemia and dysfunction due to chronic pulmonary artery obstruction and increased afterload. While cardiovascular MRI (CMR) enables noninvasive assessment of myocardial perfusion, its role in CTEPH remains unclear. The authors report adenosine stress perfusion CMR findings from two patients with CTEPH before and after pulmonary thromboendarterectomy (PTE). Both showed reduced biventricular perfusion before PTE; one demonstrated post-PTE improvement. Perfusion findings aligned with invasive hemodynamics, suggesting that CMR-derived myocardial perfusion reserve may serve as a valuable tool for assessing treatment response and RV pathophysiologic characteristics in CTEPH.

**Keywords:** Cardiac, Pulmonary Arteries, Chronic Thromboembolic Pulmonary Hypertension, Pulmonary Thromboendarterectomy, Quantitative Perfusion Cardiovascular MRI, Myocardial Blood Flow, Myocardial Perfusion Reserve

*Supplemental material is available for this article.*

© The Authors 2025. Published by the Radiological Society of North America under a CC BY 4.0 license.

**Key Points**

■ While cardiovascular MRI (CMR) enables noninvasive assessment of myocardial perfusion, its role in chronic thromboembolic pulmonary hypertension (CTEPH) remains unclear.
■ Adenosine stress perfusion CMR findings from two patients with CTEPH before and after pulmonary thromboendarterectomy showed reduced biventricular perfusion before pulmonary thromboendarterectomy (PTE), while one demonstrated post-PTE improvement but not the other.
■ CMR-derived myocardial perfusion reserve may serve as a valuable tool for assessing treatment response and right ventricular pathophysiologic characteristics in CTEPH.
■ Perfusion results were consistent with the invasive pulmonary hemodynamics both before and after PTE.

## Introduction

Chronic thromboembolic pulmonary hypertension (CTEPH) is a distinct type of PH that results from chronic obstruction of pulmonary arteries due to incompletely resolved organized thrombi usually developing after an acute pulmonary embolism (1). This chronic thrombotic obstruction frequently increases pulmonary vascular resistance (PVR) as well as pulmonary arterial pressures. In CTEPH, the right ventricle (RV) is particularly susceptible to ischemia due to supply-demand mismatch. As RV afterload increases, the RV hypertrophies and increases contractility to maintain a normal stroke volume. This compensatory mechanism requires an increase in blood flow (demand) which the RV, over time, cannot provide due, in part, to reduction of blood flow during systole and vasculature rarefaction (supply) (2). Consequently, CTEPH can lead to progressive RV and left ventricular (LV) dysfunction and ultimately heart failure and death (3–5).

Recently, cardiovascular MRI (CMR) has emerged as a promising noninvasive alternative for diagnosing PH by offering a comprehensive structural and functional assessment of the entire cardiopulmonary system in a single examination (6–8). Specifically, CMR is capable of noninvasively assessing myocardial blood flow (MBF) and myocardial perfusion reserve (MPR), which are not accessible through conventional hemodynamic measurements. These perfusion metrics offer additional insight into RV ischemia, which is thought to play a key role in the progression from adaptive to maladaptive remodeling in PH (9–12). In patients with pulmonary arterial hypertension (PAH), CMR-derived MPR index has shown to be correlated with pulmonary hemodynamics (12).

Despite its promise, the role of myocardial perfusion assessment in CTEPH remains unknown. Additionally, it is unclear whether pulmonary thromboendarterectomy (PTE), the established standard treatment for CTEPH, can improve both LV and RV perfusion. In this report, we present findings from two patients with CTEPH who underwent adenosine stress perfusion CMR before and after PTE. Both cases exhibited reduced biventricular perfusion before PTE, with improvement in the LV and RV perfusion after PTE in one of the cases but not the other. Nevertheless, the LV and RV perfusion results were found to be consistent with the invasive pulmonary hemodynamics before and after PTE, supporting the potential of CMR-derived MPR as a complementary tool to monitor treatment response and better understand RV pathophysiologic characteristics in CTEPH. CMR protocols and further details can be found in Appendix S1.

## Case Reports

### Case 1

A 52-year-old undomiciled male patient with obesity and a history of nicotine dependence and polysubstance abuse presented to an outside hospital with shortness of breath and lower extremity edema. He was diagnosed with acute decompensated heart failure and hypoxic respiratory failure. Transthoracic echocardiography (TTE) revealed mild concentric hypertrophy of the LV with normal contractility, a severely dilated RV with markedly diminished contractility, a severely dilated right atrial cavity, interventricular septal flattening throughout the cardiac cycle, and severe tricuspid regurgitation. The estimated RV systolic pressure at TTE was approximately 90 mm Hg. A pulmonary ventilation-perfusion scan was ordered due to iodinated contrast agent allergy and indicated a high probability of acute pulmonary embolism. He was started on anticoagulation and symptoms improved.

The patient later underwent coronary angiography and right heart catheterization (RHC) which revealed angiographically normal coronary arteries but severe PH due to CTEPH. He was medically managed with riociguat and anticoagulation. Six months later, the patient underwent another ventilation-perfusion scan and TTE with similar results. RHC was repeated (Table S1) demonstrating severe pre- and postcapillary PH (eg, PVR of 8.6 Wood units). Pulmonary angiography revealed dilatation of the left main pulmonary artery and severe dilatation of the right main pulmonary artery, with occlusions and perfusion defects in various segments of both lungs. Given these findings, the patient underwent PTE without complication. Follow-up included an RHC approximately 3 months after PTE which revealed improvement in pulmonary hemodynamics (eg, reduced PVR of 4.8 Wood units) (Table S1). A follow-up pulmonary angiography revealed reduction in thrombotic burden.

Approximately 1 month before PTE and 1 year after PTE, the patient underwent adenosine stress perfusion CMR for research purposes. Please refer to Appendix S1 for more details of the CMR research scan and the postprocessing. As shown in Figure A, biventricular MBF and MPR at stress were significantly reduced before PTE. Following PTE, myocardial perfusion in both the RV and LV was improved, corresponding with a reduction in the invasive pulmonary hemodynamics. For functional parameters (Figure, B), both ventricle volumes were reduced after performing PTE, especially RV volumes.

**Figure 1:**
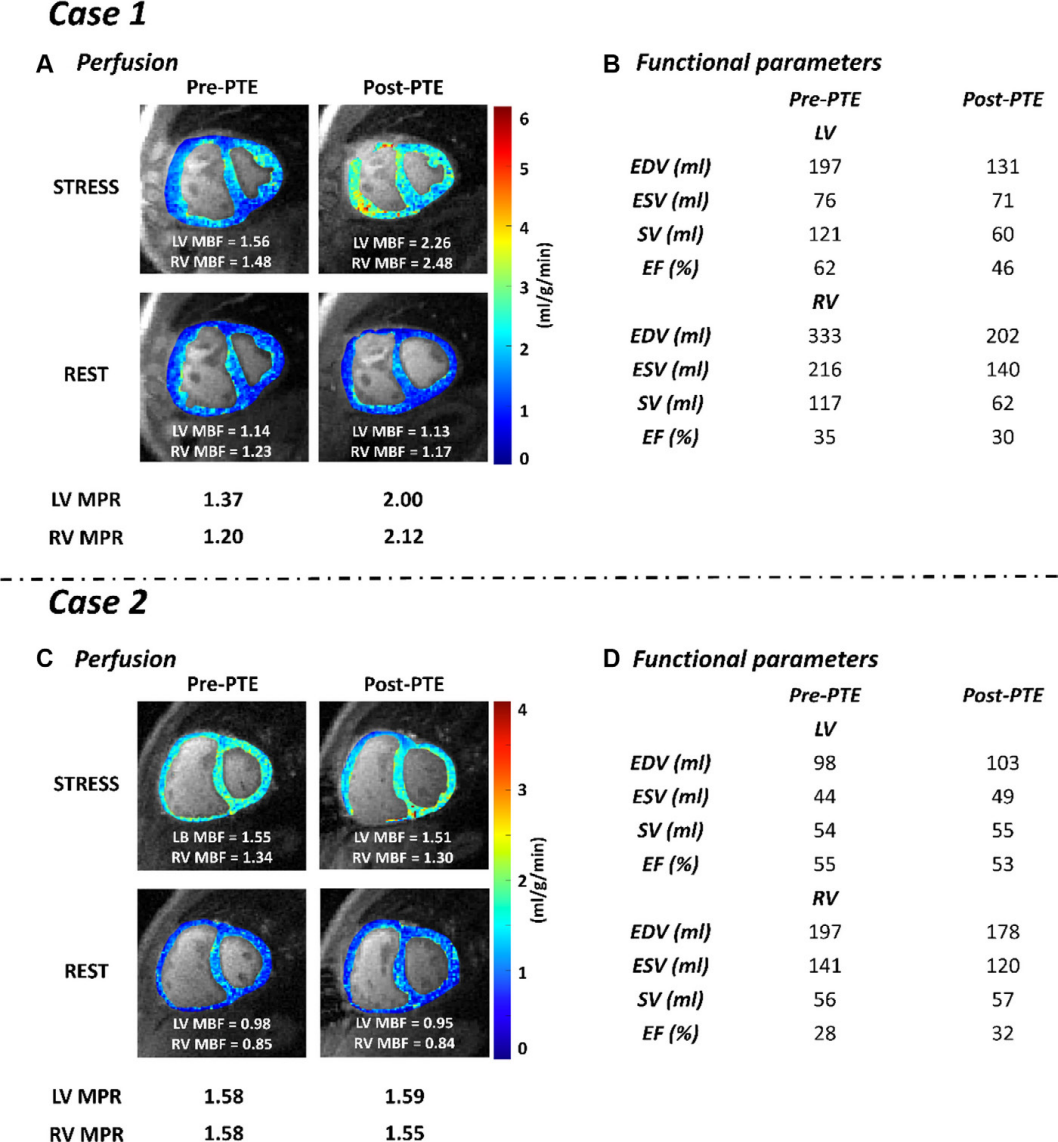
**(A)** Stress-rest MBF maps and the MPR values of case 1 before and after PTE. **(B)** Corresponding biventricular functional parameters (EDV, ESV, SV, EF) of case 1. **(C)** Stress-rest MBF maps and the MPR values of case 2 before and after PTE. **(D)** Corresponding biventricular functional parameters of case 2. EDV = end-diastolic volume, EF = ejection fraction, ESV = end-systolic volume, LV = left ventricular, MBF = myocardial blood flow, MPR = myocardial perfusion reserve, PTE = pulmonary thromboendarterectomy, RV = right ventricular, SV = stroke volume.

### Case 2

A 62-year-old woman with obesity and a past medical history of type 2 diabetes mellitus, hypertension, hypothyroidism, stroke, and chronic kidney disease presented to an outside health care facility with dry cough, fatigue, and shortness of breath. She was initially diagnosed with community-acquired pneumonia but did not improve with multiple courses of antibiotics and corticosteroids. Therefore, a TTE was performed, results of which showed an LV ejection fraction of 50%–55% with predominantly systolic intraventricular septal flattening, severely dilated and dysfunctional RV, mild tricuspid valve regurgitation, and severely elevated pulmonary arterial systolic pressure.

The patient underwent a helical CT pulmonary angiography which showed a weblike filling defect near the origin of the left upper lobe pulmonary artery, tortuous pulmonary arteries with areas of decreased perfusion, right atrial enlargement, RV hypertrophy, and contrast material reflux into the hepatic veins. Overall, the findings were consistent with CTEPH. Invasive coronary catheterization and RHC revealed angiographically normal coronary arteries but moderate to severe precapillary PH with PVR of 16.5 Wood units (Table S1). Pulmonary angiography demonstrated segmental and subsegmental chronic thrombotic disease with a subsegmental perfusion defect pattern in both lungs. She was started on riociguat and anticoagulation given her low cardiac output, marked elevation of PVR, and severe functional limitation.

The patient later underwent PTE given ongoing elevated right-sided pressures despite medical therapy. After the procedure, the patient experienced prolonged respiratory insufficiency for which she underwent RHC-PA angiography that revealed no identifiable surgical or percutaneous therapies to improve her oxygenation or decrease pulmonary pressures. Follow-up included a RHC 4 months after PTE which showed no significant change (Table S1).

Approximately 1 month before PTE and 1 year after PTE, the patient underwent adenosine stress perfusion CMR for research purposes. Stress biventricular MBF and MPR were significantly reduced before PTE. Unlike case 1, PTE did not lead to an improvement in either LV or RV perfusion as shown in Figure C. Similarly, pulmonary hemodynamics and functional parameters did not improve after PTE (Figure, D).

## Discussion

We report stress perfusion CMR findings from two patients with CTEPH who underwent PTE. Prior PTE, both patients exhibited reduced biventricular myocardial perfusion compared with a control group who underwent an identical CMR perfusion protocol (stress LV MBF = 2.66 mL/g/min; stress RV MBF = 2.56 mL/g/min; LV MPR = 2.32; RV MPR = 2.50), as reported in Fan et al (13). These findings are aligned with previous studies in patients with PAH, where impaired myocardial perfusion has been associated with disease severity (2,12,14). Following PTE, one patient showed marked improvement in both LV and RV perfusion, while the other exhibited persistently reduced perfusion. These imaging findings corresponded well with changes in invasive pulmonary hemodynamics obtained via RHC, supporting the clinical relevance of CMR perfusion metrics in this context.

To our knowledge, this is the first report of successful pixel-wise quantification of both LV and RV free-wall perfusion using advanced MRI techniques (eg, undersampled radial k-space sampling, compressed sensing image reconstruction) in patients with CTEPH before and after PTE and demonstrating its relationship with pulmonary hemodynamics.

RV ischemia is increasingly recognized as a key factor in the progression of RV dysfunction in patients with severe PAH (15) and provides distinctly different information from invasive hemodynamics. The ability to noninvasively assess RV perfusion using adenosine stress CMR in CTEPH offers a novel window into the pathophysiologic characteristics of the disease. By investigating the associations between perfusion, function, and together with pulmonary hemodynamics, we may gain new mechanistic insights into the pathophysiologic characteristics, which may eventually aid in prognosis, treatment timing, and overall disease management, warranting further study in larger patient cohorts.

## Supplemental Files

Appendix S1, Table S1

Conflicts of Interest
