# Supplementary material for: Cardiovascular MRI–based Biventricular Perfusion Assessment in Two Patients with Chronic Thromboembolic Pulmonary Hypertension Undergoing Pulmonary Thromboendarterectomy
Source: Radiol Cardiothorac Imaging. 2025 Nov 20;7(6):e250296. doi: 10.1148/ryct.250296 (PMC12728512; doi:10.1148/ryct.250296)
Supplement: Appendix S1, Table S1 [file ryct250296suppa1.pdf]

Supplemental materials undergo peer review but are not copyedited, and may include typographical errors.

©RSNA, 2025
10.1148/ryct.250296

Appendix S1

CMR protocol

MRI Hardware

The stress-rest CMR perfusion research scan was performed on a 1.5T whole-body MRI scanner (MAGNETOM Aera, Siemens Healthineers, Erlangen, Germany) equipped with a gradient system capable of achieving a maximum strength of 45 mT/m and maximum slew rate of 200 T/m/s. MRI signal reception was made using anterior and posterior coil arrays with 30 total coil elements.

Sequences parameters

Relevant imaging parameters for standard breath-hold clinical cine included: field of view (FOV) in the frequency-encoding direction ranging from 359 to 380 mm, FOV in the phase-encoding direction ranging from 285 to 363 mm, image acquisition matrix ranging from 208 × 133 to 208 x 178, spatial resolution ranging from 1.73 mm x 1.73 mm to 1.83 mm x 1.83 mm, slice thickness = 6 mm, no slice gap, TE/TR = 1.2/2.7 ms, flip angles ranging from 55° to 61°, receiver bandwidth = 925 Hz/pixel.

Relevant imaging parameters for our 2D multi-slice cardiac perfusion pulse sequence using gradient echo readout with radial k-space sampling included: FOV = 384 mm x 384 mm, matrix size =192 x 192, spatial resolution = 2 mm x 2 mm, slice thickness = 8 mm, TE/TR=1.5/2.8 ms, flip angle = 15º, minimum TS = 10 ms, B1-insensitive hybrid pulse train as the saturation pulse, 42 radial spokes per frame (corresponding to an acceleration factor of 4.6), single-shot readout duration per frame = 118 ms, 100 repetitions, electrocardiogram triggering every heartbeat. The patient was instructed to breathe normally during scanning. Each perfusion scan was performed with administration of 0.075 mmol/kg of gadobutrol (Gadavist, Bayer HealthCare Whippany, USA) at 3 mL/s using a power injector. For stress imaging, adenosine (0.14 mg/kg/min) was administered for at least 3 minutes to achieve vasodilation of coronary arteries.

Cardiac Functional Parameters

Cardiac contours were segmented using the automatic artificial intelligence tools followed by manual correction in Circle CVI42 (v5.13.10, Cardiovascular Imaging, Canada). Volumetric parameters including end-diastolic volume (EDV), end-systolic volume (ESV), stroke

Supplemental materials undergo peer review but are not copyedited, and may include typographical errors.

volume (SV) and ejection fraction (EF) were recorded. Trabeculae and papillary muscles were excluded as recommended.

Myocardial perfusion reserve (MPR) quantification

The arterial input function (AIF) and tissue function images were reconstructed from the same k-space data, but with different k-space weighted image contrast (KWIC) filters. For AIF, images were reconstructed using a KIWC filter to maintain the center of the first radial spoke only (i.e., effective TS = 10 ms) to linearize the relationship between signal intensity and gadolinium concentration. For TF, images were reconstructed using a KWIC filter to exclude the first 13 radial spokes and maintain the center of the last five radial spoke only (i.e., effective TS = 119.2 ms) to maximize the signal-to-noise ratio (SNR). Pixel-wise stress-rest myocardial blood flow (MBF) maps and the corresponding MPR were quantified for the RV free wall using the following steps: motion correction, signal normalization by the proton density weighted image, signal to T1 conversion based on the Bloch equation, T1 to gadolinium concentration ([Gd]) conversion assuming fast water exchange, T2* correction to the AIF, [Gd] to MBF conversion based on a Fermi model, rate pressure product (RPP) normalization for resting MBF, and MPR calculation as the ratio of mean stress and rest MBFs.

Supplemental materials undergo peer review but are not copyedited, and may include typographical errors.

Table S1. RHC performed pre and post PTE for both cases. Note, to keep consistency, the PVR was calculated as PVR = (mPAP - PCWP)/CO-Fick. RAP: Right atrial pressure, PASP: Pulmonary artery systolic pressure, PADP: Pulmonary artery diastolic pressure, mPAP: mean pulmonary artery pressure, SVO_2: Mixed venous oxygen saturation, PCWP: Pulmonary capillary wedge pressure, CO: Cardiac output, CI: Cardiac index, HR: Heart rate, PVR: Pulmonary vascular resistance, SV: Stroke volume.

**Right Heart Catheterization**					
	Case 1		***Case 2***		
	**1/25/2022** 90 days pre-PTE	**7/12/2022** 78 days post-PTE	**6/20/2022** 38 days pre-PTE	**11/22/2022** 117 days post-PTE	
RAP	6	1	23	24	mmHg
PASP	123	55	106	110	mmHg
PADP	40	25	40	45	mmHg
mPAP	69	39	62	64	mmHg
SVO2	66	77.5	61.8	53.9	%
PCWP	25	6	6	-	mmHg
CO - Fick	5.1	6.9	3.4	2.6	L/min
CI - Fick	2.5	3.6	1.9	1.5	$L/min/m^2$
PVR	8.6	4.8	16.5	-	Wood units
HR	73	77	93	70	Beat/min
SV	130	114.5	35.1	73.9	mL//beat
PA Compliance	1.6	3.8	0.5	1.1	mL/mmHg
